# Korean medicine use is associated with reduced lumbar surgery and opioid prescriptions in lumbar disc herniation: a nationwide cohort study

**DOI:** 10.3389/fpubh.2026.1792661

**Published:** 2026-08-07

**Authors:** Jisoo Hyun, Ho-Yeon Go, In-Hyuk Ha, Yoon Jae Lee

**Affiliations:** 1 Jaseng Hospital of Korean Medicine, Seoul, Republic of Korea; 2 National Institute for Korean Medicine Development, Gyeongsan-si, Republic of Korea; 3 Jaseng Spine and Joint Research Institute, Jaseng Medical Foundation, Seoul, Republic of Korea

**Keywords:** health insurance claims data, Korean medicine, lumbar disc herniation, lumbar surgery, opioid prescription

## Abstract

**Introduction:**

Lumbar disc herniation (LDH) is a common spinal disorder associated with chronic pain, reduced quality of life, and increased risk of lumbar surgery or long-term opioid use. Although Korean medicine treatments such as acupuncture and cupping have demonstrated effectiveness for LDH, the impact of their early intervention on surgical and opioid outcomes has not been examined at a population level. This study aimed to determine whether early Korean medicine (KM) treatment reduces the risk of lumbar surgery and opioid prescription in patients newly diagnosed with LDH compared to conventional medicine (CM) alone.

**Methods:**

We conducted a nationwide, population-based retrospective cohort study using health insurance claims data from the Health Insurance Review and Assessment Service (HIRA) of South Korea. Patients with a primary diagnosis of lumbar and other intervertebral disc disorders with radiculopathy (ICD-10: M51.1) who initiated treatment between January 1 and December 31, 2015, were included. Of 788,505 eligible patients, 1:1 propensity score matching based on sex, age group, insurance type, and Charlson Comorbidity Index yielded a surgery dataset of 121,720 patients (KM: *n* = 60,860; CM: *n* = 60,860) and an opioid dataset of 72,684 patients (KM: *n* =36,342; CM: *n* =36,342), each followed for 4 years. Cox proportional hazards regression with sequential covariate adjustment was used to estimate hazard ratios for primary outcomes of lumbar surgery and opioid analgesic prescription.

**Results:**

The KM group showed significantly lower risks of lumbar surgery (hazard ratio 0.801; 95% confidence interval, 0.762–0.842) and opioid prescription (hazard ratio 0.891 95% confidence interval, 0.844–0.940) compared to the CM group.

**Conclusion:**

These findings suggest that early KM intervention is associated with reduced rates of lumbar surgery and opioid use in LDH patients, supporting its role as an effective conservative treatment strategy.

## 1 Introduction

Lumbar disc herniation (LDH) is characterized by disruption of the annulus fibrosus, leading to displacement of the nucleus pulposus, nerve ischemia, and chemical inflammation, which cause low-back pain (LBP) and radiating pain to the lower extremities (1). LBP, the primary symptom of LDH, is a common musculoskeletal disorder, with an annual global incidence of 5–20 cases per 1,000 individuals (2) and an estimated societal cost of €8.95 billion (3). LDH- and LBP-related disabilities impose a considerable burden on individuals, healthcare systems, and social welfare systems (4), with the associated socioeconomic costs expected to increase in the future (5).

The World Federation of Neurosurgical Societies recommends conservative treatment as the first-line management for LDH, with the specific type and duration determined based on symptom severity and patient response (6). Surgical intervention is considered when symptoms persist despite at least 6–8 weeks of conservative care or in cases of progressive motor weakness, sensory deficits, or cauda equina syndrome, considering the judgment of the provider, patient characteristics, and clinical course (7). In Korea, the number of lumbar surgeries for LDH has steadily increased over time (8). Notably, 13.4% patients undergo reoperation for LDH within 5 years, with approximately half occurring within the first postoperative year (9). Moreover, the reported postoperative complication rate is 29.66% (10). These statistics highlight the clinical and public health urgency of identifying effective conservative strategies that could reduce reliance on surgical intervention. Concerns regarding overdiagnosis and unnecessary treatment of spinal disorders have also been raised in Korea, particularly in relation to early and excessive surgical intervention for LDH (11).

In addition, the use of opioid analgesics for chronic LDH-associated LBP considerably contributes to public health concerns. The opioid crisis, characterized by the widespread overprescription, addiction, and overdose of opioid analgesics, has become a major global health concern, particularly in the United States (12). In South Korea, although overall opioid consumption remains lower than in the United States, opioid prescribing for musculoskeletal and chronic pain has risen over the past decade (8), underscoring the local relevance of identifying effective conservative alternatives. A large randomized controlled trial found that opioids provided no meaningful benefit over placebo for acute low back and neck pain while carrying significant risks of harm (13). Moreover, updated clinical guidelines now explicitly recommend nonpharmacological therapies, including acupuncture, as the first-line approach for acute, subacute, and chronic LBP, reserving opioids only as a last resort when pharmacological treatment is deemed essential (14). These considerations underscore the demand for safe and effective alternatives to conventional pharmacological and surgical treatments for LDH.

Acupuncture and related therapies are increasingly recognized as conservative treatment options for spinal disorders. In Korea, integrative Korean medicine (KM) treatments—such as acupuncture and cupping—have demonstrated significant long-term effects on pain reduction, functional recovery, and quality of life in LDH patients (15). A nationwide cohort study further reported that acupuncture was associated with reduced lumbar surgery rates in patients with LBP (16). However, prior research has not examined KM intervention specifically in imaging-confirmed LDH. Unlike non-specific LBP, LDH confirmed by imaging (ICD-10: M51.1) involves structural nerve root compression, which is associated with greater severity, higher surgical rates, and increased opioid demand—suggesting that findings from general LBP populations may not be directly applicable to this distinct clinical entity. Furthermore, opioid prescription as an outcome has not been examined in prior population-based KM research. Therefore, this study aimed to evaluate the effect of early KM treatment on the risks of lumbar surgery and opioid prescription in patients newly diagnosed with LDH using nationwide claims data with a 4-year follow-up. We hypothesized that early KM intervention reduces lumbar surgery rates and opioid analgesic prescriptions in patients with LDH. The hypothesized relationships among the exposure, confounders, and outcomes are illustrated in a conceptual framework (Supplementary Figure 1).

## 2 Materials and Methods

We analyzed Nationwide Health Insurance (NHI) claims data. The study population comprised patients with a primary diagnosis of lumbar and other intervertebral disc disorders with radiculopathy (G55.1^*^) (M51.1), who received treatment for LDH between January 1 and December 31, 2015. The database contained comprehensive healthcare utilization records between January 1, 2014, and December 31, 2020, based on the NHI claims data.

This study was exempted from review by an institutional review board of the requirement for informed consent was waived because only de-identified, publicly available data were analyzed. All procedures adhered to the principles of the Declaration of Helsinki. The use of public claims data through the appropriate national data access process was conducted with approval, and the study complied with all relevant guidelines and regulations.

### 2.1 Patient and public involvement

Patients and the public were not involved in the design, conduct, reporting, or dissemination of this research. This study was based on a retrospective analysis of de-identified nationwide health insurance claims data, and no direct patient contact occurred.

### 2.2 Study population

**Figure 1:**
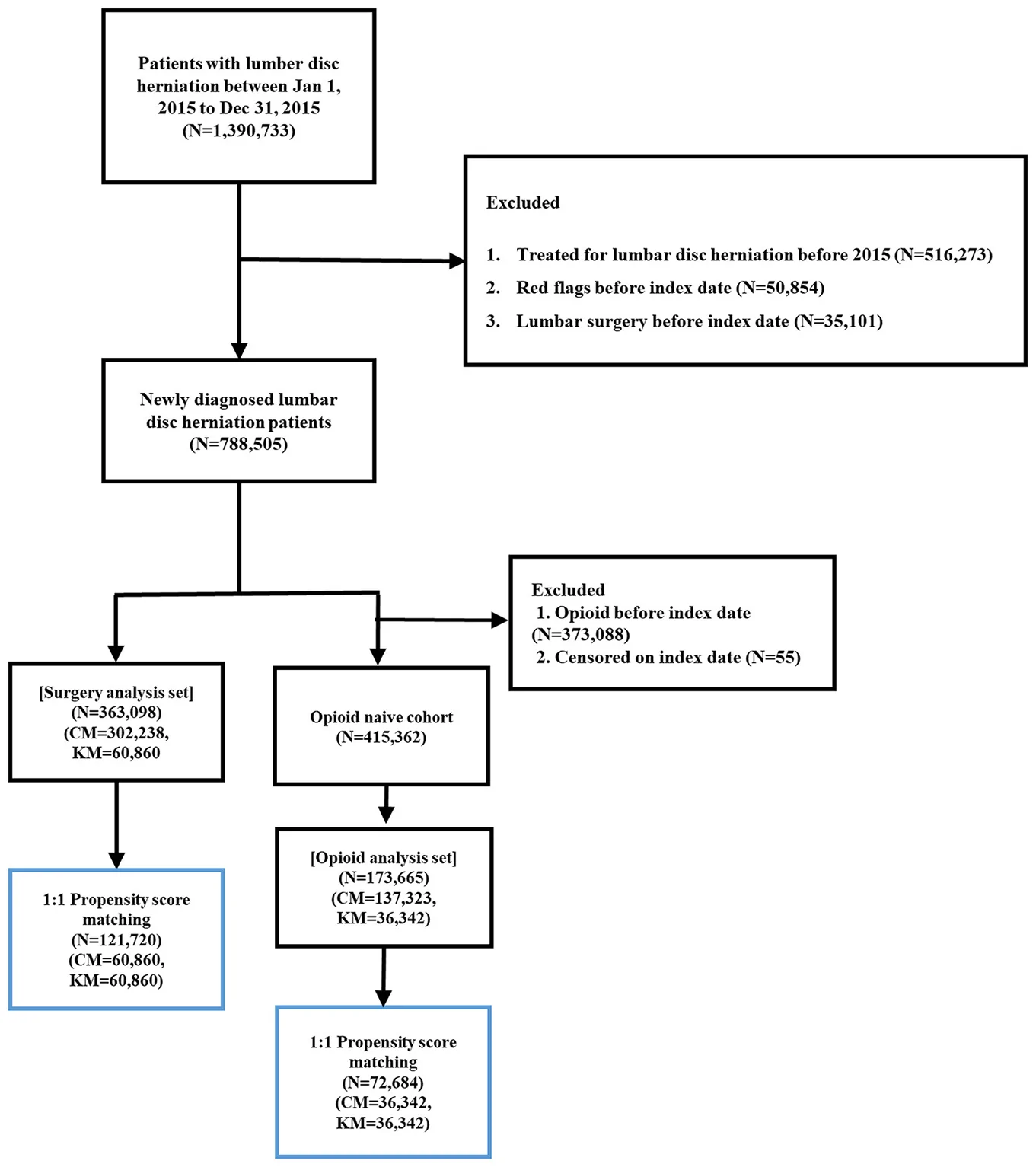
Overview of the study design for the surgery dataset **(A)** and opioid dataset **(B)**. The entry date was defined as the first diagnosis of lumbar disc herniation (LDH; ICD-10: M51.1) in 2015, and the index date was set at 60 days after the entry date. Exclusion Assessment Window 1 covers prior LDH diagnoses (M51); Exclusion Assessment Window 2 covers red flag conditions and prior lumbar surgery; Exclusion Assessment Window 3 (opioid dataset only) covers prior opioid prescriptions for low back pain or other conditions. The Exposure Assessment Window captures Korean medicine (KM) and conventional medicine (CM) treatment utilization between the entry and index dates. The Follow-up Window extends from the index date to a maximum of 4 years (December 31, 2020), during which the primary outcomes of lumbar surgery **(A)** and opioid prescription **(B)** were assessed. LDH, lumbar disc herniation; KM, Korean Medicine; CCI, Charlson Comorbidity Index, NHI, National Health Insurance.

The NHI program in Korea provides universal health coverage, with approximately 97.1% of the population enrolled as of December 2022 (17). Therefore, studies using NHI claims data may be representative of most of the Korean population. The study population included patients newly diagnosed with lumbar and other intervertebral disc disorders with radiculopathy (G55.1^*^) (M51.1) between January 1 and December 31, 2015. The entry date was defined as the date of the first diagnosis of M51.1 in 2015, whereas the index date was set at 60 days after the entry date. The 60-day exposure window was defined to be longer than the 6-week window used in a prior nationwide cohort study of acupuncture for LBP (16), reflecting the greater clinical severity and structural complexity of imaging-confirmed LDH, which may require a longer period to establish a primary treatment pattern. This window was determined in consultation with licensed Korean medicine clinicians experienced in spinal disorders. Patients were excluded if they had been diagnosed with other intervertebral disc disorders (M51) between January 1, 2014, and the entry date; had a diagnosis of “red flag” conditions (including malignancies, infections, and major trauma; Supplementary Table S1); had a history of lumbar surgery before the index date; or had been prescribed opioid analgesics for LBP or other conditions between the entry date and a day before the index date in the opioid analgesic analysis dataset (Figure 1). Missing data were not imputed, as NHI claims data represent administratively complete records of all reimbursed healthcare services; therefore, missing values were not expected for the primary variables of interest. The Charlson Comorbidity Index (CCI) was calculated for each patient based on the International Classification of Diseases (10th revision) codes, as described by Quan et al. (18).

### 2.3 Group definition

Participants were classified into two groups: KM, comprising patients with ≥ three outpatient visits to KM institutions (more than conventional medicine [CM] visits) within 60 days of the entry date; CM, comprising patients with ≥ three outpatient visits to CM institutions (without KM visits) within 60 days of the entry date. The threshold of ≥3 visits was applied to ensure that group assignment reflected sustained engagement with the respective treatment modality, rather than incidental or single-episode care, consistent with minimum session criteria used in prior claims-based KM research (16, 19).

### 2.4 Outcome measures

The primary outcomes were lumbar surgery (discectomy, laminectomy, or fusion) and opioid analgesic prescription. An outcome event was confirmed if a patient underwent lumbar surgery or received an opioid prescription during this period. The follow-up period was defined as the duration from the index date (60 days after the entry date) to a maximum of 4 years. Lumbar surgery was defined as open discectomy (including laminectomy), endoscopic discectomy (including laminectomy), laminectomy of the lumbar spine, or spinal fusion. The corresponding procedure codes for each surgery are summarized in Supplementary Table S2.

Opioid prescription was defined as ≥14 days of tramadol prescription or ≥7 days of non-tramadol opioid prescription (20, 21). The 7-day threshold for non-tramadol opioids was adopted based on evidence that opioid use extending beyond 3–7 days in acute pain settings is not associated with additional clinical benefit and may worsen functional outcomes (22). The 14-day threshold for tramadol was informed by established opioid prescribing guidelines (23) and observational studies employing empirically derived exposure definitions (24). Two opioid-related outcomes were assessed: the prescription of tramadol plus non-tramadol opioids and the prescription of non-tramadol opioids only. Opioid analgesics were defined as medications under the Anatomical Therapeutic Chemical classification code N02A, with the corresponding drug names and codes presented in Supplementary Table S3.

In the opioid analysis dataset, patients were censored if they received an opioid prescription for conditions other than LBP, were diagnosed with a “red flag” condition, or underwent lumbar surgery before the opioid outcome event during follow-up or before the occurrence of the primary outcome.

### 2.5 Statistical analysis

Categorical variables, including patient demographics, healthcare utilization for LBP-related diagnoses, and the CCI, are presented as frequencies and percentages, whereas continuous variables are presented as means and standard deviations. Between-group differences (KM vs. CM) were assessed using the chi-square or Fisher's exact test for categorical variables and the independent *t*-test for continuous variables.

Propensity score matching (PSM) was performed to balance potential confounders and minimize selection bias when comparing lumbar surgery and opioid prescription outcomes between groups. Propensity scores were estimated using logistic regression, with a caliper width of 0.1 and 1:1 nearest-neighbor matching. The PSM model included sex, age group, insurance type, and CCI as covariates. Comorbidity variables were excluded from the PSM model to prevent overfitting and to preserve common support between groups, given the substantial disparity in group sizes (CM: *n* = 302,238; KM: *n* = 60,860) and the large number of potential comorbidity covariates. Inclusion of all comorbidity variables in the propensity score model would have substantially reduced the matched sample size and compromised the representativeness of the analytic cohort. Instead, comorbidity variables and healthcare utilization for LBP-related diagnoses were adjusted for in the subsequent Cox regression models, representing a two-stage confounding control approach recognized in large administrative dataset analyses (25). Healthcare utilization was categorized into quartiles and included across all models as a proxy for disease severity, given the substantial differences observed between groups before and after PSM. Healthcare utilization was used as a proxy for disease severity, as patients with more frequent healthcare visits may reflect greater symptom burden or disease complexity that could influence the likelihood of surgical intervention or opioid prescription. Covariate balance after matching was evaluated using standardized mean differences, with an absolute standardized mean difference > 0.1 indicating imbalance.

Time-to-event analyses for lumbar surgery and opioid prescription were conducted using Kaplan–Meier curves and the log-rank test. Hazard ratios (HRs) with 95% confidence intervals (CIs) were estimated using Cox proportional hazards regression. Treatment exposure was defined as a fixed variable based on the treatment pattern observed during the 60-day exposure assessment window prior to the index date. Model fit was assessed using the Akaike Information Criterion (AIC) across sequential Cox models with increasing covariate adjustment (Models 1–4). The proportional hazards assumption was evaluated using the Schoenfeld residual test. To quantify the potential impact of unmeasured confounding, E-values were calculated for the primary hazard ratio estimates using the method described by VanderWeele and Ding (26). To examine whether the findings were sensitive to the definition of the exposure assessment window, we additionally analyzed an alternative 1-year exposure assessment window using the same modeling approach (Supplementary Tables S6–S7). All analyses were performed using SAS Enterprise Guide version 7.1 (SAS Institute, Cary, NC, USA), with a two-sided significance level of 0.05.

## 3 Results

### 3.1 Study population and dataset composition

**Figure 2:**
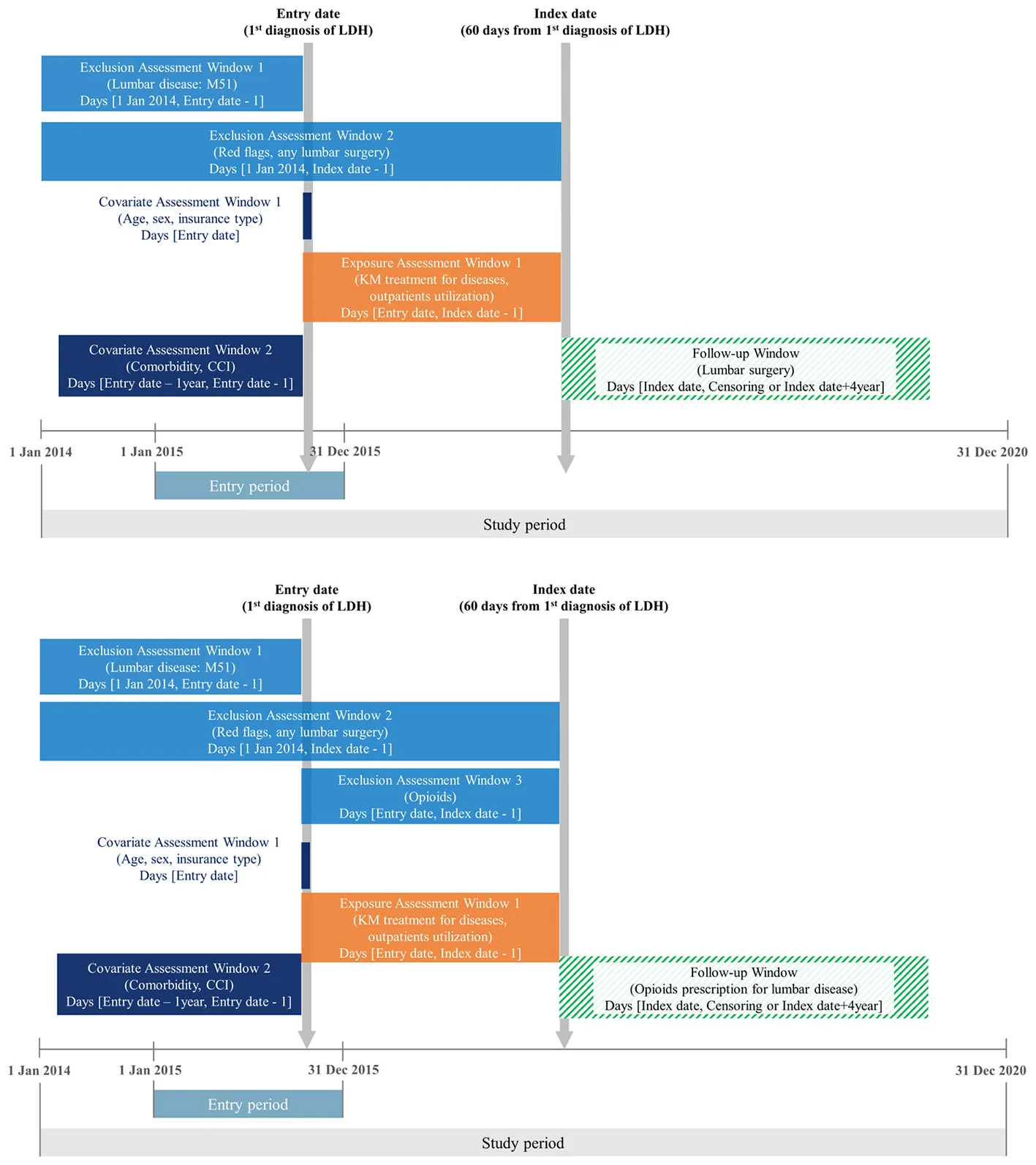
Flowchart of patient selection for the surgery and opioid analytic datasets. Of 1,390,733 patients diagnosed with LDH (ICD-10: M51.1) between January 1 and December 31, 2015, 788,505 met the eligibility criteria after exclusions for prior LDH diagnosis (*n* =516,273), red flag conditions (*n* = 50,854), and prior lumbar surgery (*n* = 35,101). The surgery dataset comprised 363,098 patients (KM: *n* = 60,860; CM: *n* = 302,238). For the opioid dataset, patients with prior opioid prescriptions (*n* =373,088) and those censored on the index date (*n* = 55) were additionally excluded, yielding an opioid-naïve cohort of 415,362 patients, from which 173,665 were eligible (KM: *n* = 36,342; CM: *n* = 137,323). After 1:1 propensity score matching, the final surgery dataset included 121,720 matched patients (KM: *n* = 60,860; CM: *n* = 60,860) and the opioid dataset included 72,684 matched patients (KM: *n* = 36,342; CM: *n* = 36,342). CM, conventional medicine; KM, Korean medicine; PSM, propensity score matching.

Between January 1 and December 31, 2015, 1,390,733 patients were diagnosed with lumbar and other intervertebral disc disorders with radiculopathy (G55.1^*^) (M51.1). Of these, 788,505 patients met the eligibility criteria and were classified into the KM and CM groups. The surgery dataset comprised 363,098 patients (KM: *n* =60,860; CM: *n* = 302,238). In the opioid dataset, patients who had received opioid prescriptions for LBP or other conditions from the entry date to 1 day before the index date were excluded, yielding 173,665 patients (KM: *n* = 36,342; CM: *n* = 137,323; Figure 2).

### 3.2 Baseline characteristics before and after PSM

In the surgery and opioid datasets, significant differences were observed between groups in sex, age, insurance type, and CCI before PSM. The KM group had a higher proportion of women and patients aged ≥ 60 years, greater proportion of NHI beneficiaries, and tendency toward higher CCI scores than the CM group. Therefore, 1:1 PSM was performed based on sex, age group, insurance type, and CCI, to address these differences, yielding 60,860 and 36,342 patients in each group in the surgery and opioid datasets, respectively. After matching, no significant differences were observed in demographic and clinical characteristics, and all variables had standardized mean differences of 0.00, indicating well-balanced baseline characteristics (Table 1). Comorbidity variables not included in the PSM model showed some residual differences after matching, as did healthcare utilization variables (Supplementary Table S4). Comorbidity variables were subsequently adjusted for in the Cox regression models as part of the two-stage confounding control approach.

**Table 1:** Baseline characteristics of the study population.

Variables	Before propensity score matching						After propensity score matching					
	CM		KM		*p*–value	SMD	CM		KM		*p*–value	SMD
	N	%	N	%			N	%	N	%		
Surgery dataset	302,238		60,860				60,860		60,860			
Sex												
Male	129,342	42.8	25,761	42.3	0.034	0.01	25,761	42.3	25,761	42.3	1.000	0.00
Female	172,896	57.2	35,099	57.7		−0.01	35,099	57.7	35,099	57.7		0.00
Age group, years												
< 50	117,826	39.0	23,458	38.5	< 0.001	0.01	23,458	38.5	23,458	38.5	1.000	0.00
50–59	75,874	25.1	13,904	22.9		0.05	13,904	22.9	13,904	22.9		0.00
60–69	58,763	19.4	11,958	19.7		−0.01	11,958	19.7	11,958	19.7		0.00
70–79	39,840	13.2	9,439	15.5		−0.07	9,439	15.5	9,439	15.5		0.00
≥80	9,935	3.3	2,101	3.5		−0.01	2,101	3.5	2,101	3.5		0.00
Insurance type												
NHI	287,259	95.0	58,819	96.7	< 0.001	−0.08	58,819	96.7	58,819	96.7	1.000	0.00
Medical aid	14,979	5.0	2,041	3.4		0.08	2,041	3.4	2,041	3.4		0.00
CCI												
0	197,320	65.3	39,570	65.0	0.473	0.01	39,570	65.0	39,570	65.0	1.000	0.00
1	73,039	24.2	14,787	24.3		0.00	14,787	24.3	14,787	24.3		0.00
2	21,205	7.0	4,294	7.1		0.00	4,291	7.1	4,291	7.1		0.00
≥3	10,674	3.5	2,212	3.6		−0.01	2,212	3.6	2,212	3.6		0.00
Opioid dataset	137,323		36,342				36,342		36,342			
Sex												
Male	57,809	42.1	15,461	42.5	0.126	−0.01	15,461	42.5	15,461	42.5	1.000	0.00
Female	79,514	57.9	20,881	57.5		0.01	20,881	57.5	20,881	57.5		0.00
Age group, years												
< 50	60,255	43.9	15,250	42.0	< 0.001	0.04	15,250	42.0	15,250	42.0	1.000	0.00
50–59	34,059	24.8	8,243	22.7		0.05	8,243	22.7	8,243	22.7		0.00
60–69	24,555	17.9	6,776	18.7		−0.02	6,776	18.7	6,776	18.7		0.00
70–79	14,954	10.9	4,992	13.7		−0.09	4,992	13.7	4,992	13.7		0.00
≥80	3,500	2.6	1,081	3.0		−0.03	1,081	3.0	1,081	3.0		0.00
Insurance type												
NHI	132,034	96.2	35,331	97.2	< 0.001	−0.06	35,331	97.2	35,331	97.2	1.000	0.00
Medical aid	5,289	3.8	1,011	2.8		0.06	1,011	2.8	1,011	2.8		0.00
CCI												
0	94,564	68.9	24,674	67.9	0.001	0.02	24,674	67.9	24,674	67.9	< 0.001	0.00
1	30,940	22.5	8,360	23.0		−0.01	8,360	23.0	8,360	23.0		0.00
2	8,086	5.9	2,236	6.2		−0.01	2,236	6.2	2,236	6.2		0.00
≥3	3,733	2.7	1,072	3.0		−0.01		3.0	1,072	3.0		0.00

CM, Conventional Medicine; KM, Korean Medicine; CCI, Charlson Comorbidity Index; SMD, Standardized Mean Difference; NHI, National Health Insurance.

### 3.3 Lumbar surgery outcomes after matching

**Figure 3:**
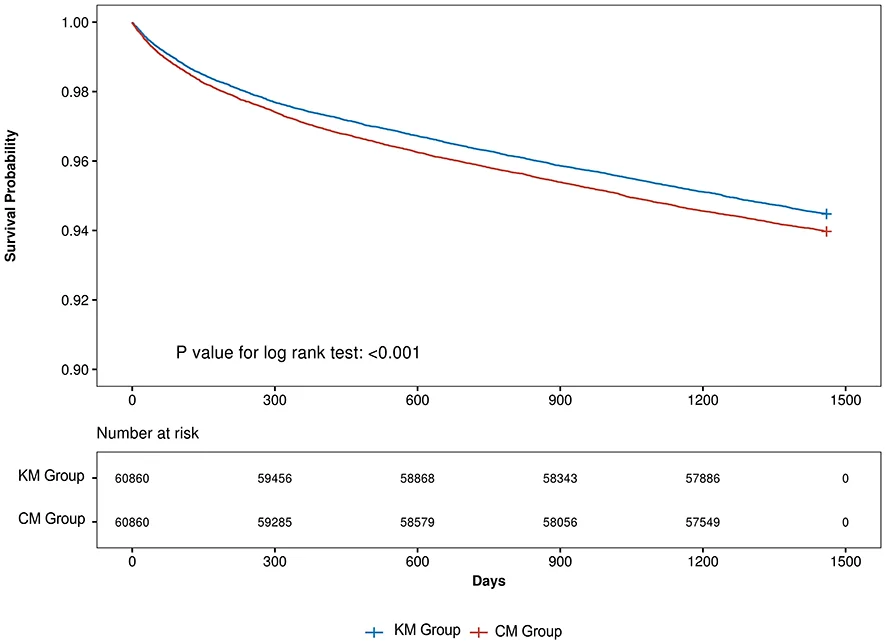
Kaplan–Meier survival curves for the cumulative incidence of lumbar surgery in the Korean medicine (KM) and conventional medicine (CM) groups after 1:1 propensity score matching. The y-axis represents the probability of remaining free from lumbar surgery (survival probability), and the x-axis represents follow-up time in days from the index date (60 days after the initial LDH diagnosis). The blue line labeled “KM group” represents the KM group (*n* = 60,860) and the red line labeled “CM group” represents the CM group (*n* = 60,860). The number-at-risk table below the curve shows the number of patients remaining at risk at each time point. The log-rank test indicated a statistically significant difference in surgery-free survival between groups (*p* < 0.001). CM, conventional medicine; KM, Korean medicine; LDH, lumbar disc herniation.

In the matched cohorts, the incidence of lumbar surgery was compared between the KM and CM groups from the index date (60 days of the entry date) up to a maximum follow-up of 4 years using Kaplan–Meier survival curves (Figure 3). During follow-up, 3,362 (5.52%) and 3,669 (6.03%) patients in the KM and CM groups, respectively, underwent lumbar surgery representing an absolute risk difference of 0.51 percentage points [number needed to treat (NNT) = 196]. The log-rank test indicated a significant difference in surgery incidence between groups (*p* < 0.001, Supplementary Table S5), with the KM group consistently demonstrating lower cumulative surgery incidence throughout the follow-up period.

Because the Schoenfeld residual test indicated that the proportional hazards assumption was violated for the surgery outcome across all models, both before and after PSM (*p* < 0.001, Supplementary Table S9), time-stratified hazard ratios at 1-year and 4-year follow-up are presented as the primary results for this endpoint, rather than a single pooled estimate (Table 2). In the fully adjusted model (Model 4), early KM treatment was associated with a lower risk of lumbar surgery at both time points: the HR was 0.715 (95% CI, 0.665–0.769) at 1 year, corresponding to a 28.5% relative hazard reduction (absolute risk difference 0.37 percentage points; NNT = 270), and 0.801 (95% CI, 0.762–0.842) at 4 years, corresponding to a 19.9% relative hazard reduction. The association remained statistically significant across all sequential Cox models (Models 1–4), with model fit improving progressively as indicated by decreasing AIC values (Supplementary Table S10).

**Table 2:** Univariate and multivariate logistic regression analyses of surgery and opioid use between Korean and conventional medicine user groups.

Variables	Surgery−1-year follow–up (after PSM)				Surgery−4-year follow–up (after PSM)				Opioid−4-year follow–up (after PSM)			
	M1	M2	M3	M4	M1	M2	M3	M4	M1	M2	M3	M4
Group (ref. CM)	0.718 (0.668–0.771)^***^	0.720 (0.670–0.774)^***^	0.721 (0.671–0.774)^***^	0.715 (0.665–0.769)^***^	0.794 (0.755–0.834)^***^	0.801 (0.762–0.841)^***^	0.801 (0.763–0.842)^***^	0.801 (0.762–0.842)^***^	0.889 (0.843–0.937)^***^	0.894 (0.848–0.943)^***^	0.895 (0.848–0.944)^***^	0.891 (0.844–0.940)^***^
Healthcare utilization (ref. Q1)												
Q2 (26–50%)	1.223 (1.093–1.367)^***^	1.222 (1.093–1.367)^***^	1.220 (1.091–1.365)^***^	1.195 (1.068–1.336)^**^	1.168 (1.084–1.258)^***^	1.163 (1.080–1.253)^***^	1.160 (1.077–1.250)^***^	1.137 (1.055–1.225)^***^	1.044 (0.955–1.140)	1.055 (0.966–1.153)	1.051 (0.962–1.148)	1.012 (0.927–1.106)
Q3 (51–75%)	1.476 (1.328–1.640)^***^	1.469 (1.322–1.633)^***^	1.466 (1.319–1.630)^***^	1.401 (1.260–1.558)^***^	1.365 (1.272–1.465)^***^	1.347 (1.255–1.446)^***^	1.344 (1.252–1.442)^***^	1.285 (1.197–1.379)^***^	1.121 (1.047–1.201)^**^	1.122 (1.047–1.202)^**^	1.118 (1.044–1.197)^**^	1.062 (0.992–1.138)
Q4 (76–100%)	2.184 (1.983–2.406)^***^	2.125 (1.929–2.342)^***^	2.120 (1.924–2.336)^***^	1.956 (1.773–2.156)^***^	1.746 (1.634–1.865)^***^	1.665 (1.558–1.779)^***^	1.660 (1.553–1.773)^***^	1.535 (1.436–1.642)^***^	1.248 (1.166–1.335)^***^	1.184 (1.107–1.268)^***^	1.180 (1.103–1.263)^***^	1.074 (1.003–1.151)^*^
Sex (ref. Male)	—	0.684 (0.639–0.733)^***^	0.686 (0.640–0.735)^***^	0.642 (0.597–0.691)^***^	—	0.748 (0.713–0.784)^***^	0.750 (0.715–0.787)^***^	0.699 (0.665–0.735)^***^	—	0.917 (0.870–0.966)^**^	0.921 (0.874–0.971)^**^	0.817 (0.773–0.863)^***^
Age, years (ref. < 50)												
50–59	—	1.189 (1.081–1.308)^***^	1.170 (1.063–1.288)^**^	1.020 (0.924–1.125)	—	1.420 (1.328–1.520)^***^	1.392 (1.301–1.490)^***^	1.220 (1.138–1.308)^***^	—	2.308 (2.136–2.494)^***^	2.245 (2.077–2.426)^***^	1.925 (1.779–2.084)^***^
60–69	—	1.626 (1.484–1.782)^***^	1.574 (1.432–1.730)^***^	1.181 (1.066–1.310)^**^	—	2.070 (1.941–2.207)^***^	1.987 (1.860–2.123)^***^	1.512 (1.408–1.625)^***^	—	3.561 (3.304–3.838)^***^	3.370 (3.122–3.639)^***^	2.468 (2.275–2.677)^***^
70–79	—	1.544 (1.398–1.707)^***^	1.475 (1.328–1.637)^***^	0.990 (0.879–1.115)	—	2.104 (1.965–2.253)^***^	1.988 (1.851–2.135)^***^	1.357 (1.252–1.471)^***^	—	4.561 (4.224–4.926)^***^	4.237 (3.913–4.588)^***^	2.733 (2.503–2.984)^***^
≥80	—	0.821 (0.647–1.042)	0.781 (0.615–0.993)^*^	0.540 (0.422–0.691)^***^	—	0.935 (0.792–1.104)	0.880 (0.744–1.041)	0.615 (0.518–0.731)^***^	—	1.369 (1.207–1.553)^***^	4.042 (3.554–4.598)^***^	2.631 (2.301–3.009)^***^
Payer type (ref. NHI)	—	0.807 (0.657–0.991)^*^	0.789 (0.642–0.969)^*^	0.734 (0.597–0.902)^**^	—	0.900 (0.789–1.028)	0.877 (0.768–1.001)	0.821 (0.719–0.938)^**^	—	1.369 (1.207–1.553)^***^	1.333 (1.175–1.512)^***^	1.271 (1.120–1.441)^***^
CCI (ref. 0)												
1	—	—	1.035 (0.953–1.123)	0.987 (0.908–1.073)	—	—	1.071 (1.012–1.132)^*^	1.018 (0.962–1.077)	—	—	1.155 (1.088–1.225)^***^	1.079 (1.016–1.145)^*^
2	—	—	1.189 (1.048–1.348)^**^	1.103 (0.971–1.252)	—	—	1.200 (1.102–1.306)^***^	1.104 (1.013–1.203)^*^	—	—	1.279 (1.169–1.400)^***^	1.144 (1.044–1.254)^**^
≥3	—	—	1.233 (1.046–1.453)^*^	1.109 (0.939–1.310)	—	—	1.287 (1.155–1.434)^***^	1.142 (1.023–1.275)^*^	—	—	1.336 (1.184–1.509)^***^	1.140 (1.009–1.289)^*^
Comorbid conditions (ref. No)												
Spondylolisthesis	—	—	—	1.753 (1.508–2.039)^***^	—	—	—	1.680 (1.513–1.865)^***^	—	—	—	1.522 (1.353–1.711)^***^
Scoliosis	—	—	—	0.834 (0.567–1.228)	—	—	—	0.878 (0.676–1.140)	—	—	—	1.076 (0.836–1.386)
Other arthrosis	—	—	—	1.038 (0.957–1.126)	—	—	—	1.044 (0.988–1.103)	—	—	—	1.279 (1.208–1.354)^***^
Rheumatoid arthritis	—	—	—	1.168 (0.954–1.431)	—	—	—	1.317 (1.158–1.498)^***^	—	—	—	1.368 (1.182–1.583)^***^
Osteoporosis	—	—	—	1.132 (1.004–1.275)^*^	—	—	—	1.091 (1.009–1.181)^*^	—	—	—	1.147 (1.060–1.242)^***^
Mental disorder	—	—	—	0.953 (0.867–1.047)	—	—	—	0.984 (0.924–1.048)	—	—	—	1.172 (1.098–1.251)^***^
Other deforming dorsopathies	—	—	—	1.115 (0.791–1.572)	—	—	—	1.171 (0.928–1.478)	—	—	—	1.332 (1.038–1.709)^*^
Spondylosis	—	—	—	1.166 (1.065–1.276)^***^	—	—	—	1.154 (1.085–1.228)^***^	—	—	—	1.260 (1.180–1.347)^***^
Other spondylopathies	—	—	—	1.974 (1.817–2.144)^***^	—	—	—	1.904 (1.800–2.014)^***^	—	—	—	1.836 (1.729–1.949)^***^
Dorsalgia	—	—	—	1.295 (1.198–1.401)^***^	—	—	—	1.279 (1.212–1.350)^***^	—	—	—	1.276 (1.206–1.351)^***^
Biomechanical lesions	—	—	—	1.071 (0.817–1.404)	—	—	—	1.111 (0.928–1.330)	—	—	—	1.375 (1.151–1.643)^***^
Dislocation	—	—	—	1.070 (0.995–1.151)	—	—	—	1.008 (0.958–1.061)	—	—	—	1.001 (0.946–1.060)

^1^Model 1: Adjusted for healthcare utilization (categorized into quartiles).

^2^Model 2: Adjusted for healthcare utilization (quartiles) and demographic variables (age, sex, and health insurance type).

^3^Model 3: Adjusted for healthcare utilization, demographic variables, and CCI.

^4^Model 4: Adjusted for healthcare utilization, demographic variables, CCI, and comorbidities.

NHI, National Health Insurance; CCI, Charlson Comorbidity Index; HR, hazard ratio; CI, confidence interval; CM, Conventional Medicine; KM, Korean Medicine.

^*^p-value < 0.05, ^**^p-value < 0.01, ^***^p-value < 0.001.

The statistical violation of the proportional hazards assumption may also partly reflect the high statistical power inherent to the large sample size.

### 3.4 Opioid prescription outcomes after matching

In the matched cohorts, the incidence of opioid prescriptions (opioids, ≥7 days; tramadol, ≥14 days) between the index date and after 4 years was compared between groups using Kaplan–Meier survival analysis (Supplementary Figure 2). During follow-up, 2,957 and 3,103 patients in the KM and CM groups, respectively, received opioid analgesic prescriptions, representing an absolute risk difference of 0.40 percentage points (NNT =250). The log-rank test revealed a statistically significant difference between groups (*p* = 0.016, Supplementary Table S5).

Over a 4-year follow-up, early KM treatment was associated with a significantly lower risk of opioid prescription. The HR for receiving any opioid prescription, including tramadol ≥14 days, was 0.891 (95% CI, 0.844–0.940) in the fully adjusted model (Model 4), indicating a 10.9% relative reduction in the hazard of opioid prescription (Table 2). The association remained consistent across all adjusted Cox models, with AIC values indicating progressive improvement in model fit (Supplementary Table S10). The proportional hazards assumption was satisfied for the composite opioid outcome (including tramadol) after PSM across all models (*p* = 0.133–0.222, Supplementary Table S9), supporting the validity of the Cox regression estimates. For the non-tramadol opioid outcome, the Schoenfeld test indicated violation after PSM (*p* < 0.001), which should be interpreted with caution given the relatively small number of events (*n* = 1,020).

For non-tramadol opioid prescriptions, 485 (1.33%) and 535 (1.47%) patients in the KM and CM groups, respectively, received prescriptions during follow-up. The log-rank test did not reach statistical significance (*p* = 0.102, Supplementary Table S5); however, after full adjustment in the Cox regression model (Model 4), the HR was 0.823 (95% CI, 0.721–0.939), indicating a statistically significant 17.7% relative hazard reduction (Supplementary Table S8). In sensitivity analyses using an alternative 1-year exposure assessment window, the associations were materially unchanged for both lumbar surgery (fully adjusted HR 0.805; 95% CI, 0.766–0.845) and opioid prescription (HR 0.835; 95% CI, 0.776–0.898), indicating that the findings were not an artifact of the 60-day exposure-window definition (Supplementary Tables S6–S7).

## 4 Discussion

LDH is a major spinal disorder affecting approximately 2–3% of the global population (27). Although most cases improve with conservative treatment, unnecessary surgeries are sometimes performed (28). In addition, the inappropriate use of opioid analgesics for pain management (12) and association between opioid use and increased risk of falls (29), dementia (30), and mortality (31) in older adults have raised substantial medical and social concerns.

In this study, we investigated whether early KM treatment in patients with newly diagnosed LDH is associated with subsequent reductions in lumbar surgery rates and opioid analgesic prescriptions. Our findings indicated that early KM treatment was associated with a reduced risk of lumbar surgery and fewer opioid prescriptions. These associations may reflect the combined benefits of KM as a composite intervention, encompassing acupuncture (32), cupping (33), and other modalities for LDH, as well as the neuroprotective and anti-inflammatory actions of herbal medicine and pharmacopuncture (34). As the present study evaluated KM as a composite exposure based on claims data, the relative contributions of individual treatment modalities cannot be determined. In addition, previous randomized controlled trials have shown that nonpharmacological KM interventions such as acupuncture, electroacupuncture, cupping, and Chuna are associated with greater improvements than pharmacologic treatment for LDH (35), which may have reduced the analgesic medication needs in our study population.

The time-varying nature of the treatment effect for the surgery outcome—stronger at 1 year than at 4 years—warrants interpretation. This pattern may indicate that early KM intervention primarily delays rather than permanently prevents surgery in a subset of patients whose underlying structural pathology eventually requires surgical management. It is also possible that patients in the CM group who avoided early surgery represent a self-selected subgroup with milder disease that similarly avoids surgery over the longer term, narrowing the between-group difference over time. Clinically, this suggests that the principal benefit of early KM intervention lies in reducing or delaying surgery during the acute-to-subacute period—a benefit with meaningful implications for cost, recovery time, and patient preference—even if long-term surgical risk converges between groups.

Although a previous nationwide cohort study examined the effect of acupuncture on lumbar surgery rates in patients with LBP (16), to our knowledge, no nationwide, population-based study has evaluated the association between comprehensive KM interventions and the rates of lumbar surgery and opioid prescription in patients with LDH. Notably, the HR for lumbar surgery in patients with LBP who received KM treatment in our study (0.801) is higher than that reported in a previous nationwide cohort study (0.633) (16), indicating a more modest relative risk reduction in the present study. This disparity may be explained by differences in patient populations: the previous study included patients with general LBP, whereas our study focused exclusively on patients with a confirmed LDH diagnosis—a population with greater disease severity and a higher likelihood of requiring surgery, which may have attenuated the relative effect of KM intervention—as well as methodological differences including a longer follow-up period (4 years vs. 2 years), a more comprehensive KM exposure definition encompassing multiple modalities, and the inclusion of healthcare utilization as a covariate in the regression models. In the opioid analysis, a stronger protective association was observed when tramadol was excluded from the composite outcome (HR 0.823 vs. HR 0.891). This may be because tramadol is not classified as a controlled narcotic substance in Korea and is therefore prescribed more liberally than strictly regulated opioids, potentially diluting the between-group difference when included in the composite outcome.

The clinical significance of these findings warrants careful consideration. Although the absolute risk differences were modest (0.51 percentage points for surgery; 0.40 percentage points for opioid prescription), these should be interpreted in the context of the naturally low baseline surgical rate in LDH—a condition in which the majority of patients recover with conservative care (CM group 4-year surgery rate: 6.03%). In this setting, relative hazard reductions of 19.9% for surgery and 10.9% for opioid prescription represent meaningful effects concentrated among patients who would otherwise progress to surgical intervention or long-term opioid use. Furthermore, given the scale of LDH incidence in Korea (788,505 newly diagnosed patients in 2015 alone), even modest individual-level reductions may translate into a substantial public health impact at the population level.

These findings may also inform health policy. The association of early KM intervention with lower rates of surgery and opioid prescription supports considering KM as an explicit early option within stepped-care pathways for LDH, and provides population-level evidence relevant to coverage decisions for KM services within the national health insurance system. Prospective and economic evaluations would help determine whether broader early access to KM care is cost-effective at the system level.

The temporal pattern of the treatment effect also provides important clinical insight. The more pronounced hazard reduction at 1-year follow-up (HR 0.715) compared with 4 years (HR 0.801) suggests that the protective association of early KM intervention is strongest during the acute and subacute phases of LDH. This pattern is consistent with the natural history of the condition, wherein the critical window for conservative intervention lies in the early clinical course. The attenuation of the effect over time may reflect the eventual surgical conversion of patients with more severe or refractory disease, regardless of initial treatment modality. These findings underscore the importance of timely KM intervention in newly diagnosed LDH patients. These findings contribute to the theoretical framework supporting early conservative intervention for LDH, providing population-level evidence that timely KM treatment may help redirect the clinical trajectory away from surgical and pharmacological management, particularly during the acute and subacute phases of the condition.

To assess the robustness of our findings to potential unmeasured confounding, E-values were calculated (Supplementary Table S11). The E-value for lumbar surgery (HR 0.801) was 1.804, and that for opioid prescription (HR 0.891) was 1.492, indicating that an unmeasured confounder would need to be associated with both KM treatment and the respective outcome by a risk ratio of at least 1.804- and 1.492-fold, respectively, to fully explain away the observed associations. These values suggest that a moderately strong unmeasured confounder — beyond those already adjusted for through PSM and sequential covariate adjustment — would be required to negate the observed findings. While unmeasured confounding such as health-seeking behavior or lifestyle factors cannot be entirely excluded, it is unlikely that such factors would be sufficiently strong to fully account for the observed associations, particularly for the surgery outcome.

This study has some notable strengths. First, it utilized nationwide claims data from the NHI, encompassing the entire Korean population and reflecting real-world data from actual clinical practice. This facilitated a reliable analysis without self-reporting bias and enabled long-term follow-up (up to 4 years) without attrition owing to patient dropout or hospital switching. Second, in Korea, various KM treatments such as acupuncture, cupping, moxibustion, and electroacupuncture are covered under NHI, enabling claims data to provide valuable evidence on their utilization, effectiveness, and safety. Third, real-world data encompasses a more heterogeneous patient population with varying ages, comorbidities, and concomitant treatments than randomized control trials, which may have limited generalizability, thereby offering a more accurate reflection of real-world clinical practice. The findings of this study may therefore inform clinical decision-making and healthcare policy development.

This study has several limitations that should be acknowledged. First, disease severity could not be fully assessed for comparability between groups. Surgical decision-making for LDH involves multiple clinical factors, including the degree and direction of disc herniation, pain severity, neurological deficits, and bowel/bladder dysfunction, which were not available in the Health Insurance Review and Assessment Service (HIRA) claims data. This absence of clinical variables introduces the possibility of indication bias and residual confounding, whereby unmeasured differences in disease severity between groups may have influenced both treatment selection and outcomes. The calculated E-values suggest that a moderately strong unmeasured confounder would be required to fully explain the observed associations; nonetheless, this limitation cannot be entirely ruled out. In particular, healthcare utilization was used as a proxy for disease severity, but this proxy is imperfect: KM care inherently involves more frequent outpatient visits than conventional pharmacologic care, independent of underlying severity. Utilization may therefore partly capture treatment modality rather than severity, and the observed associations may reflect, in part, baseline severity differences between groups rather than the effect of KM treatment alone. The E-value analysis should be interpreted in this light—while a moderately strong confounder would be required to fully explain away the effect, severity-related confounding could plausibly approach that magnitude in this clinical context. Second, the operational definition of the KM and CM groups based on ≥3 outpatient visits within 60 days may introduce selection bias. Patients who sought early KM care may systematically differ from those receiving CM alone in terms of health beliefs, healthcare-seeking behavior, socioeconomic factors, or symptom severity. Although PSM was applied to balance measured baseline characteristics, unmeasured differences between groups may persist. In addition, requiring KM visits to outnumber CM visits for KM-group assignment excluded patients who received substantial combined KM–CM care. This was intended to define modality-dominant groups for a clearer contrast, but it limits generalizability to real-world practice, in which many patients use both modalities concurrently; the present findings therefore apply most directly to patients whose early care was predominantly one modality. Third, since the HIRA database includes only reimbursed services, non-reimbursed KM treatments, such as herbal medicine and pharmacopuncture, could not be identified; similar limitations may apply to the CM group. This may result in underestimation of the true KM exposure and its effects. Because such non-reimbursed services would, if captured, increase the measured intensity of KM exposure in the KM group, this misclassification would most plausibly bias the observed association toward the null, leading to an underestimation rather than an overestimation of the true effect of KM treatment. Fourth, comorbidity variables were excluded from the PSM procedure to prevent overfitting and preserve common support, given the substantial disparity in group sizes and the large number of potential covariates. Although some comorbidity variables remained imbalanced after matching, additional adjustments were made in regression models, and the direction of imbalance—with variables such as Dorsalgia and Dislocation more prevalent in the KM group—would be expected to bias results toward the null, potentially underestimating the true protective effect of KM treatment. Fifth, treatment exposure was defined as a fixed variable based on the 60-day exposure assessment window. Treatment crossover or changes in treatment modality during the follow-up period were not accounted for in the primary analysis, which may have introduced misclassification of exposure over time. Sixth, surgical decisions for LDH may be influenced by physician recommendations even in mild cases, while some patients with severe disease may opt for continued conservative care. Moreover, opioid prescriptions are not obligatory and may vary based on patient preference, which could not be accounted for in the analysis. Seventh, we defined opioid exposure as ≥7 days for opioids or ≥14 days for tramadol; however, long-term opioid use was not evaluated. Finally, the generalizability of these findings beyond the Korean healthcare context warrants careful consideration. Korea has a unique healthcare infrastructure in which KM is fully integrated into the national health insurance system and widely accepted culturally. In settings where KM is less accessible, reimbursed, or culturally accepted, the observed associations may not be directly applicable.

Therefore, future studies combining HIRA claims data with electronic medical record data from individual institutions and prospective clinical studies are needed to evaluate the long-term effects of KM treatment on surgery rates and opioid use in patients with LDH of similar clinical severity. Studies employing time-varying exposure definitions and richer clinical data would also help address the methodological limitations of the present study. Such studies would enhance the clinical applicability of these findings.

In conclusion, using claims data from the HIRA, this nationwide, population-based study investigated the association between early KM treatment following LDH diagnosis and subsequent lumbar surgery and opioid analgesic prescriptions. Early KM intervention was significantly associated with reduced hazards of lumbar surgery (HR 0.801; 95% CI, 0.762–0.842) and opioid prescriptions (HR 0.891; 95% CI, 0.844–0.940) over a 4-year follow-up period. These findings suggest that early KM treatment for LDH is associated with reduced rates of lumbar surgery and opioid analgesic use, providing clinically relevant evidence for healthcare professionals and policymakers. However, given the observational design of this study, causal inference cannot be established, and further prospective studies are warranted to validate these findings.

## Data Availability

The data analyzed in this study is subject to the following licenses/restrictions: The datasets analyzed in this study are subject to restrictions imposed by the Health Insurance Review and Assessment Service (HIRA) of Korea. The data are not publicly available due to legal and ethical restrictions related to personal data protection and data use agreements. Access to the data is granted only through formal application and approval by HIRA for specific research purposes. Requests to access these datasets should be directed to Yoon Jae Lee, goodsmile8119@gmail.com.
