# Supplementary material for: Korean medicine use is associated with reduced lumbar surgery and opioid prescriptions in lumbar disc herniation: a nationwide cohort study
Source: Front Public Health. 2026 Aug 7;14:1792661. doi: 10.3389/fpubh.2026.1792661 (PMC13493509; doi:10.3389/fpubh.2026.1792661)
Supplement: Supplementary file 1 [file Table_1.docx]

Supplementary Tables

Table S1. Red flags

| CODE | Full name |
| --- | --- |
| C00-C97 | Malignant neoplasms |
| V01-V99 | Transport accidents |
| D16.6 | Benign neoplasm of the vertebral column |
| D32.1 | Benign neoplasm of the spinal meninges |
| D33.4 | Benign neoplasm of the spinal cord |
| M45 | Ankylosing spondylitis |
| M86 | Osteomyelitis |
| S32 | Fracture of the lumbar spine and pelvis |
| G06.1 | Intraspinal abscess and granuloma |
| M46.0 | Spinal enthesopathy |
| M46.9 | Inflammatory spondylopathy, unspecified |
| M89.6 | Osteopathy after poliomyelitis |
| M90.2 | Osteopathy in other infectious diseases classified elsewhere |
| T09.3 | Injury of the spinal cord, level unspecified |
| T08.1 | Fracture of the spine, level unspecified, open |
| S34.1 | Other injuries of the lumbar spinal cord |
| S34.3 | Injury to the cauda equina |
