# Supplementary material for: Korean medicine use is associated with reduced lumbar surgery and opioid prescriptions in lumbar disc herniation: a nationwide cohort study
Source: Front Public Health. 2026 Aug 7;14:1792661. doi: 10.3389/fpubh.2026.1792661 (PMC13493509; doi:10.3389/fpubh.2026.1792661)
Supplement: Supplementary file 2 [file Table_10.docx]

**Table S10. Akaike Information Criterion (AIC) values across Cox regression models**

| Outcome |  | Model 1 | Model 2 | Model 3 | Model 4 |
| --- | --- | --- | --- | --- | --- |
| Surgery (4-year) | Before PSM | 540,848 | 538,354 | 538,180 | 535,140 |
|  | After PSM | 163,935 | 163,181 | 163,154 | 162,215 |
| Surgery (1-year) | Before PSM | 256,791 | 255,991 | 255,954 | 254,393 |
|  | After PSM | 77,643 | 77,421 | 77,415 | 76,943 |
| Opioid (including tramadol) | Before PSM | 336,106 | 331,254 | 331,140 | 328,748 |
|  | After PSM | 133,734 | 131,676 | 131,631 | 130,630 |
| Opioid (excluding tramadol) | Before PSM | 56,502 | 55,699 | 55,653 | 55,215 |
|  | After PSM | 22,674 | 22,298 | 22,290 | 22,109 |
