# Supplementary material for: Korean medicine use is associated with reduced lumbar surgery and opioid prescriptions in lumbar disc herniation: a nationwide cohort study
Source: Front Public Health. 2026 Aug 7;14:1792661. doi: 10.3389/fpubh.2026.1792661 (PMC13493509; doi:10.3389/fpubh.2026.1792661)
Supplement: Supplementary file 3 [file Table_11.docx]

**Table S11. E-values for observed associations between early Korean medicine treatment and primary outcomes**

| **Outcome** | **HR (95% CI)** | **E-value (point estimate)** | **E-value (95% CI upper bound)** |
| --- | --- | --- | --- |
| Lumbar surgery | 0.801 (0.762–0.842) | 1.804 | 1.661 |
| Opioid prescription (including tramadol) | 0.891 (0.844–0.940) | 1.492 | 1.325 |
| Opioid prescription (excluding tramadol) | 0.823 (0.721–0.939) | 1.697 | 1.328 |
