# Supplementary material for: Korean medicine use is associated with reduced lumbar surgery and opioid prescriptions in lumbar disc herniation: a nationwide cohort study
Source: Front Public Health. 2026 Aug 7;14:1792661. doi: 10.3389/fpubh.2026.1792661 (PMC13493509; doi:10.3389/fpubh.2026.1792661)
Supplement: Supplementary file 4 [file Table_2.docx]

**Table S2.** Procedure codes and names for lumbar surgery

| CODE | Full name |
| --- | --- |
| N1493 | Diskectomy (invasive)-lumbar spine |
| N1494 | Diskectomy by endoscopy |
| N1499 | Laminectomy, lumbar spine |
| N2499 | Laminectomy, lumbar spine |
| N0466 | Arthrodesis of the lumbar spine-anterior technique |
| N1466 | Arthrodesis of the lumbar spine-anterior technique |
| N0469 | Arthrodesis of the lumbar spine-posterior technique |
| N1469 | Arthrodesis of the lumbar spine-posterior technique |
| N2470' | Posterior lumbar interbody fusion |
| N1460 | Posterior lumbar interbody fusion |
