# Supplementary material for: Korean medicine use is associated with reduced lumbar surgery and opioid prescriptions in lumbar disc herniation: a nationwide cohort study
Source: Front Public Health. 2026 Aug 7;14:1792661. doi: 10.3389/fpubh.2026.1792661 (PMC13493509; doi:10.3389/fpubh.2026.1792661)
Supplement: Supplementary file 5 [file Table_3.docx]

**Table S3.** ATC codes used for the analysis of opioid analgesics

| Drug | ATC code | Full name |
| --- | --- | --- |
| Tramadol | N02AJ13 | Tramadol and paracetamol |
|  | N02AX02 | Tramadol |
|  | N02AX52 | Tramadol, combinations |
| Opioid | N02AA01 | Morphine |
|  | N02AA03 | Hydromorphone |
|  | N02AA05 | Oxycodone |
|  | N02AA08 | Dihydrocodeine |
|  | N02AA55 | Oxycodone and naloxone |
|  | N02AB02 | Pethidine |
|  | N02AB03 | Fentanyl |
|  | N02AE01 | Buprenorphine |
|  | N02AF01 | Butorphanol |
|  | N02AF02 | Nalbuphine |
|  | N02AJ | Opioids in combination with non-opioid analgesics |
|  | N02AJ09 | Codeine and other non-opioid analgesics |
|  | N02AX06 | Tapentadol |
