# Supplementary material for: Korean medicine use is associated with reduced lumbar surgery and opioid prescriptions in lumbar disc herniation: a nationwide cohort study
Source: Front Public Health. 2026 Aug 7;14:1792661. doi: 10.3389/fpubh.2026.1792661 (PMC13493509; doi:10.3389/fpubh.2026.1792661)
Supplement: Supplementary file 6 [file Table_4.docx]

**Table S4.** Baseline characteristics by covariates not used in propensity score matching (PSM)

| Variables | Before PSM | | | | | After PSM | | | | | |  |
| --- | --- | --- | --- | --- | --- | --- | --- | --- | --- | --- | --- | --- |
|  | WM | | KM | | p-value | WM | | KM | | p-value | |  |
|  | N | % | N | % |  | N | % | N | % | |  | |
| Surgery dataset | 302,238 |  | 60,860 |  |  | 60,860 |  | 60,860 |  | |  | |
| Comorbidity |  |  |  |  |  |  |  |  |  | |  | |
| Disorders of the nerve roots and spinal cord | 2,309 | 0.8 | 442 | 0.7 | 0.328 | 460 | 0.8 | 442 | 0.7 | | 0.548 | |
| Spondylolisthesis | 6,646 | 2.2 | 1,539 | 2.5 | < 0.001 | 1,353 | 2.2 | 1,539 | 2.5 | | < 0.001 | |
| Scoliosis | 2,389 | 0.8 | 486 | 0.8 | 0.837 | 468 | 0.8 | 486 | 0.8 | | 0.558 | |
| Other arthrosis | 89,376 | 29.6 | 17,943 | 29.5 | 0.661 | 18,656 | 30.7 | 17,934 | 29.5 | | < 0.001 | |
| Rheumatoid arthritis | 6,821 | 2.3 | 1,299 | 2.1 | 0.062 | 1,373 | 2.3 | 1,299 | 2.1 | | 0.140 | |
| Osteoporosis | 24,063 | 8.0 | 5,615 | 8.7 | < 0.001 | 5,305 | 8.7 | 5,315 | 8.7 | | 0.912 | |
| Mental disorder | 46,123 | 15.3 | 9,624 | 15.8 | < 0.001 | 9,603 | 15.8 | 9,624 | 15.8 | | 0.864 | |
| Other deforming dorsopathies | 2,107 | 0.7 | 431 | 0.7 | 0.765 | 442 | 0.7 | 431 | 0.7 | | 0.708 | |
| Spondylosis | 41,998 | 13.9 | 7,893 | 13.0 | < 0.001 | 8,707 | 14.3 | 7,893 | 13.0 | | < 0.001 | |
| Other spondylopathies | 56,051 | 18.6 | 11,414 | 18.8 | 0.226 | 11,681 | 19.2 | 11,414 | 18.8 | | 0.035 | |
| Dorsalgia | 169,078 | 55.9 | 39,699 | 65.2 | < 0.001 | 34,325 | 56.4 | 39,699 | 65.2 | | < 0.001 | |
| Biomechanical lesions | 2,803 | 0.9 | 730 | 1.2 | < 0.001 | 568 | 0.9 | 730 | 1.2 | | < 0.001 | |
| Dislocation | 85,813 | 28.4 | 21,766 | 35.8 | < 0.001 | 17,074 | 28.1 | 21,765 | 35.8 | | < 0.001 | |
| Healthcare utilization |  |  |  |  | < 0.001 |  |  |  |  | | < 0.001 | |
| Q1 (0–25%) | 87,406 | 41.6 | 8,918 | 14.7 |  | 25,272 | 41.5 | 8,918 | 14.7 | |  |  |
| Q2 (26–50%) | 64,036 | 21.2 | 11,659 | 19.2 |  | 13,000 | 21.4 | 11,659 | 19.2 | |  |  |
| Q3 (51–75%) | 58,094 | 19.2 | 15,863 | 26.1 |  | 11,815 | 19.4 | 15,863 | 26.1 | |  |  |
| Q4 (76–100%) | 54,323 | 18.0 | 24,420 | 40.1 |  | 10,773 | 17.7 | 24,420 | 40.1 | |  |  |
| Opioid dataset | 137,323 |  | 36,342 |  |  | 36,342 |  | 36,342 |  | |  | |
| Comorbidity |  |  |  |  |  |  |  |  |  | |  | |
| Disorders of the nerve roots and spinal cord | 905 | 0.7 | 235 | 0.7 | < 0.001 | 241 | 0.7 | 235 | 0.7 | | 0.783 | |
| Spondylolisthesis | 2,661 | 1.9 | 889 | 2.5 | 0.795 | 706 | 1.9 | 889 | 2.5 | | < 0.001 | |
| Scoliosis | 1,136 | 0.8 | 292 | 0.8 | < 0.001 | 284 | 0.8 | 292 | 0.8 | | 0.738 | |
| Other arthrosis | 34,518 | 25.1 | 9,267 | 25.5 | 0.656 | 9,656 | 26.6 | 9,267 | 25.5 | | < 0.001 | |
| Rheumatoid arthritis | 2,548 | 1.9 | 629 | 1.7 | 0.156 | 686 | 1.9 | 629 | 1.7 | | 0.107 | |
| Osteoporosis | 9,310 | 6.8 | 2,803 | 7.7 | 0.115 | 2,739 | 7.5 | 2,803 | 7.7 | | 0.336 | |
| Mental disorder | 17,805 | 13.0 | 4,974 | 13.7 | < 0.001 | 4,944 | 13.6 | 4,974 | 13.7 | | 0.739 | |
| Other deforming dorsopathies | 850 | 0.6 | 246 | 0.7 | < 0.001 | 240 | 0.7 | 246 | 0.7 | | 0.785 | |
| Spondylosis | 15,910 | 11.6 | 4,137 | 11.4 | 0.215 | 4,339 | 11.9 | 4,137 | 11.4 | | 0.018 | |
| Other spondylopathies | 20,193 | 14.7 | 5,822 | 16.0 | 0.283 | 5,743 | 15.8 | 5,822 | 16.0 | | 0.343 | |
| Dorsalgia | 71,328 | 51.9 | 22,611 | 62.2 | < 0.001 | 19,077 | 52.5 | 22,611 | 62.2 | | < 0.001 | |
| Biomechanical lesions | 1,086 | 0.8 | 381 | 1.1 | < 0.001 | 319 | 0.9 | 381 | 1.1 | | 0.019 | |
| Dislocation | 37,942 | 27.6 | 12,629 | 34.8 | < 0.001 | 9,814 | 27.0 | 12,629 | 34.8 | | < 0.001 | |
| Healthcare utilization |  |  |  |  | < 0.001 |  |  |  |  | | < 0.001 | |
| Q1 (0–25%) | 62,899 | 45.8 | 6,350 | 17.5 |  | 16,613 | 45.7 | 6,350 | 17.5 | |  |  |
| Q2 (26–50%) | 16,810 | 12.2 | 4,240 | 11.7 |  | 4,537 | 12.5 | 4,240 | 11.7 | |  |  |
| Q3 (51–75%) | 32,106 | 23.4 | 10,748 | 29.6 |  | 8,535 | 23.5 | 10,748 | 29.6 | |  |  |
| Q4 (76–100%) | 25,508 | 18.6 | 15,004 | 41.3 |  | 6,657 | 18.3 | 15,004 | 41.3 | |  |  |
