# Supplementary material for: Korean medicine use is associated with reduced lumbar surgery and opioid prescriptions in lumbar disc herniation: a nationwide cohort study
Source: Front Public Health. 2026 Aug 7;14:1792661. doi: 10.3389/fpubh.2026.1792661 (PMC13493509; doi:10.3389/fpubh.2026.1792661)
Supplement: Supplementary file 7 [file Table_5.docx]

**Table S5.** Log-rank test (after PSM)

|  | binary | Total | Failed | Censored | P-value for Log-rank test |
| --- | --- | --- | --- | --- | --- |
| Surgery  (4-year) | WM | 60,860 | 3,669 | 57,191 | < 0.001 |
|  | KM | 60,860 | 3,362 | 57,498 |  |
|  | Total | 121,720 | 7,031 | 114,689 |  |
| Opioid | WM | 36,342 | 3,103 | 33,239 | 0.016 |
|  | KM | 36,342 | 2,957 | 33,385 |  |
|  | Total | 72,684 | 6,060 | 66,624 |  |
| Opioid (excluding tramadol) | WM | 36,342 | 535 | 35,807 | 0.102 |
|  | KM | 36,342 | 485 | 35,807 |  |
|  | Total | 72,684 | 1,020 | 71,664 |  |
