# Supplementary material for: Korean medicine use is associated with reduced lumbar surgery and opioid prescriptions in lumbar disc herniation: a nationwide cohort study
Source: Front Public Health. 2026 Aug 7;14:1792661. doi: 10.3389/fpubh.2026.1792661 (PMC13493509; doi:10.3389/fpubh.2026.1792661)
Supplement: Supplementary file 8 [file Table_6.docx]

**Table S6.** Sensitivity analysis: Cox regression for lumbar surgery using a 1-year exposure assessment window

|  | Surgery (after PSM) | | | |
| --- | --- | --- | --- | --- |
|  | Model 1^1^ | Model 2^2^ | Model 3^3^ | Model 4^4^ |
|  | HR (95% CI) | HR (95% CI) | HR (95% CI) | HR (95% CI) |
| **Group**  **(ref. CM)** | 0.774 (0.737-0.812) *** | 0.792 (0.754-0.831) *** | 0.793 (0.756-0.833) *** | 0.805 (0.766-0.845) *** |
| **Healthcare utilization**  **(ref. Q1)** |  |  |  |  |
| **Q2 (26-50%)** | 1.324 (1.218-1.440) *** | 1.290 (1.187-1.403) *** | 1.287 (1.183-1.399) *** | 1.255 (1.154-1.364) *** |
| **Q3 (51-75%)** | 1.686 (1.566-1.815) *** | 1.598 (1.484-1.721) *** | 1.591 (1.477-1.713) *** | 1.510 (1.402-1.626) *** |
| **Q4 (76-100%)** | 2.651 (2.476-2.839) *** | 2.384 (2.224-2.555) *** | 2.364 (2.205-2.534) *** | 2.117 (1.972-2.272) *** |
| **Sex**  **(ref. Male)** |  | 0.786 (0.750-0.825) *** | 0.790 (0.754-0.829) *** | 0.749 (0.713-0.787) *** |
| **Age, years (ref. < 50)** |  |  |  |  |
| 50–59 |  | 1.613 (1.508-1.725) *** | 1.565 (1.462-1.675) *** | 1.421 (1.326-1.523) *** |
| 60–69 |  | 2.326 (2.180-2.482) *** | 2.198 (2.057-2.349) *** | 1.797 (1.674-1.929) *** |
| 70–79 |  | 2.170 (2.023-2.328) *** | 2.005 (1.863-2.157) *** | 1.530 (1.411-1.658) *** |
| ≥80 |  | 0.817 (0.680-0.981) * | 0.753 (0.627-0.906) ** | 0.578 (0.480-0.698) *** |
| **Payer type (ref. NHI)** |  | 0.869 (0.764-0.988) * | 0.838 (0.736-0.954) ** | 0.800 (0.703-0.910) *** |
| **CCI (ref. 0)** |  |  |  |  |
| 1 |  |  | 1.115 (1.056-1.178) *** | 1.073 (1.015-1.134) * |
| 2 |  |  | 1.275 (1.176-1.382) *** | 1.188 (1.095-1.290) *** |
| ≥3 |  |  | 1.406 (1.267-1.560) *** | 1.270 (1.143-1.411) *** |
| **Comorbid conditions (ref. No)** |  |  |  |  |
| Spondylolisthesis |  |  |  | 1.544 (1.384-1.722) *** |
| Scoliosis |  |  |  | 1.081 (0.848-1.379) |
| Other arthrosis |  |  |  | 1.070 (1.014-1.128) * |
| Rheumatoid arthritis |  |  |  | 1.322 (1.170-1.494) *** |
| Osteoporosis |  |  |  | 1.044 (0.967-1.127) |
| Mental disorder |  |  |  | 1.045 (0.984-1.110) |
| Other deforming dorsopathies |  |  |  | 1.089 (0.851-1.394) |
| Spondylosis |  |  |  | 1.082 (1.016-1.151) * |
| Other spondylopathies |  |  |  | 1.675 (1.585-1.771) *** |
| Dorsalgia |  |  |  | 1.100 (1.045-1.159) *** |
| Biomechanical lesions |  |  |  | 1.149 (0.957-1.380) |
| Dislocation |  |  |  | 0.960 (0.912-1.011) |
| CCI, Charlson Comorbidity Index; HR, hazard ratio; CI, confidence interval; CM: Conventional Medicine; KM: Korean Medicine  *p-value < 0.05, **p-value < 0.01, ***p-value < 0.001 | | | | |
