# Supplementary material for: Korean medicine use is associated with reduced lumbar surgery and opioid prescriptions in lumbar disc herniation: a nationwide cohort study
Source: Front Public Health. 2026 Aug 7;14:1792661. doi: 10.3389/fpubh.2026.1792661 (PMC13493509; doi:10.3389/fpubh.2026.1792661)
Supplement: Supplementary file 9 [file Table_7.docx]

**Table S7.** Sensitivity analysis: Cox regression for opioid prescription using a 1-year exposure assessment window

|  | Opioid (after PSM) | | | |
| --- | --- | --- | --- | --- |
|  | Model 1^1^ | Model 2^2^ | Model 3^3^ | Model 4^4^ |
|  | HR (95% CI) | HR (95% CI) | HR (95% CI) | HR (95% CI) |
| **Group**  **(ref. CM)** | 0.808 (0.753-0.869) *** | 0.835 (0.777-0.897) *** | 0.836 (0.778-0.898) *** | 0.835 (0.776-0.898) *** |
| **Healthcare utilization**  **(ref. Q1)** |  |  |  |  |
| **Q2 (26-50%)** | 1.228 (1.103-1.367) *** | 1.203 (1.081-1.339) *** | 1.202 (1.080-1.339) *** | 1.168 (1.049-1.300) ** |
| **Q3 (51-75%)** | 1.485 (1.335-1.652) *** | 1.400 (1.259-1.557) *** | 1.394 (1.253-1.551) *** | 1.323 (1.189-1.472) *** |
| **Q4 (76-100%)** | 1.836 (1.660-2.032) *** | 1.586 (1.432-1.756) *** | 1.579 (1.426-1.748) *** | 1.428 (1.287-1.583) *** |
| **Sex**  **(ref. Male)** |  | 0.946 (0.883-1.015) | 0.952 (0.887-1.020) | 0.881 (0.819-0.947) *** |
| **Age, years (ref. < 50)** |  |  |  |  |
| 50–59 |  | 2.259 (2.054-2.484) *** | 2.190 (1.990-2.409) *** | 1.979 (1.796-2.180) *** |
| 60–69 |  | 3.172 (2.882-3.490) *** | 2.987 (2.709-3.295) *** | 2.399 (2.163-2.660) *** |
| 70–79 |  | 3.843 (3.466-4.261) *** | 3.549 (3.189-3.949) *** | 2.618 (2.332-2.940) *** |
| ≥80 |  | 3.502 (2.907-4.219) *** | 3.188 (2.638-3.851) *** | 2.326 (1.914-2.828) *** |
| **Payer type (ref. NHI)** |  | 1.212 (0.995-1.476) | 1.173 (0.963-1.428) | 1.143 (0.938-1.392) |
| **CCI (ref. 0)** |  |  |  |  |
| 1 |  |  | 1.197 (1.104-1.297) *** | 1.147 (1.058-1.244) *** |
| 2 |  |  | 1.329 (1.166-1.516) *** | 1.227 (1.074-1.402) ** |
| ≥3 |  |  | 1.346 (1.116-1.623) ** | 1.222 (1.012-1.476) * |
| **Comorbid conditions (ref. No)** |  |  |  |  |
| Spondylolisthesis |  |  |  | 1.237 (1.010-1.514) * |
| Scoliosis |  |  |  | 0.915 (0.617-1.358) |
| Other arthrosis |  |  |  | 1.274 (1.177-1.379) *** |
| Rheumatoid arthritis |  |  |  | 1.084 (0.857-1.372) |
| Osteoporosis |  |  |  | 1.116 (0.991-1.257) |
| Mental disorder |  |  |  | 1.248 (1.137-1.371) *** |
| Other deforming dorsopathies |  |  |  | 1.510 (1.064-2.141) * |
| Spondylosis |  |  |  | 1.185 (1.072-1.310) *** |
| Other spondylopathies |  |  |  | 1.597 (1.463-1.743) *** |
| Dorsalgia |  |  |  | 1.202 (1.119-1.292) *** |
| Biomechanical lesions |  |  |  | 1.325 (0.992-1.771) |
| Dislocation |  |  |  | 0.916 (0.847-0.992) * |
| CCI, Charlson Comorbidity Index; HR, hazard ratio; CI, confidence interval; CM: Conventional Medicine; KM: Korean Medicine  *p-value < 0.05, **p-value < 0.01, ***p-value < 0.001 | | | | |
