# Supplementary material for: Korean medicine use is associated with reduced lumbar surgery and opioid prescriptions in lumbar disc herniation: a nationwide cohort study
Source: Front Public Health. 2026 Aug 7;14:1792661. doi: 10.3389/fpubh.2026.1792661 (PMC13493509; doi:10.3389/fpubh.2026.1792661)
Supplement: Supplementary file 10 [file Table_8.docx]

Table S8. Cox regression (opioids excluding tramadol)

|  | Opioid excluding tramadol (after PSM) | | | |  |
| --- | --- | --- | --- | --- | --- |
|  | Model 1^1^ | Model 2^2^ | Model 3^3^ | Model 4^4^ | |
|  | HR (95% CI) | HR (95% CI) | HR (95% CI) | HR (95% CI) | |
| **Group**  **(ref. CM)** | 0.828  (0.727-0.943) ** | 0.836  (0.734-0.952) ** | 0.837  (0.735-0.953) ** | 0.823  (0.721-0.939) ** | |
| **Healthcare utilization (ref. Q1)** |  |  |  |  | |
| **Q2 (26-50%)** | 1.168  (0.945-1.444) | 1.182  (0.956-1.461) | 1.175  (0.951-1.453) | 1.129  (0.914-1.396) | |
| **Q3 (51-75%)** | 1.106  (0.931-1.313) | 1.099  (0.925-1.306) | 1.094  (0.921-1.299) | 1.036  (0.872-1.232) | |
| **Q4 (76-100%)** | 1.424  (1.208-1.678) *** | 1.340  (1.137-1.580) *** | 1.334  (1.131-1.573) *** | 1.201  (1.017-1.419) * | |
| **Sex**  **(ref. Male)** |  | 0.880  (0.775-1.000) * | 0.887  (0.781-1.007) | 0.792  (0.693-0.906) *** | |
| **Age, years (ref. < 50)** |  |  |  |  | |
| 50–59 |  | 2.067  (1.697–2.519) ^***^ | 1.996  (1.636–2.435) ^***^ | 1.713  (1.400-2.097) *** | |
| 60–69 |  | 3.401  (2.821-4.100) *** | 3.176  (2.624-3.845) *** | 2.317  (1.891-2.838) *** | |
| 70–79 |  | 5.096  (4.235-6.133) *** | 4.651  (3.837-5.636) *** | 2.981  (2.410-3.686) *** | |
| ≥ 80 |  | 5.042  (3.777-6.731) *** | 4.530  (3.373-6.083) *** | 2.926  (2.147-3.986) *** | |
| **Payer type (ref. NHI)** |  | 1.358  (1.012-1.822) * | 1.310  (0.976-1.760) | 1.247  (0.928-1.675) | |
| **CCI (ref. 0)** |  |  |  |  | |
| 1 |  |  | 1.165  (1.008-1.347) * | 1.087  (0.939-1.258) | |
| 2 |  |  | 1.318  (1.065-1.632) * | 1.167  (0.941-1.448) | |
| ≥3 |  |  | 1.517  (1.160-1.985) ** | 1.284  (0.978-1.685) | |
| **Comorbid conditions (ref. No)** |  |  |  |  | |
| Spondylolisthesis |  |  |  | 1.453  (1.100-1.917) ** | |
| Scoliosis |  |  |  | 0.967  (0.518-1.806) | |
| Other arthrosis |  |  |  | 1.124  (0.978–1.292) | |
| Rheumatoid arthritis |  |  |  | 1.286  (0.912-1.814) | |
| Osteoporosis |  |  |  | 1.175  (0.976–1.416) | |
| Mental disorder |  |  |  | 1.229  (1.055-1.432) ** | |
| Other deforming dorsopathies |  |  |  | 1.205  (0.645-2.250) | |
| Spondylosis |  |  |  | 1.219  (1.041-1.428) * | |
| Other spondylopathies |  |  |  | 1.979  (1.717-2.282) *** | |
| Dorsalgia |  |  |  | 1.409  (1.221-1.626) *** | |
| Biomechanical lesions |  |  |  | 1.403  (0.940-2.095) | |
| Dislocation |  |  |  | 1.064  (0.929-1.220) | |
