# Supplementary material for: Korean medicine use is associated with reduced lumbar surgery and opioid prescriptions in lumbar disc herniation: a nationwide cohort study
Source: Front Public Health. 2026 Aug 7;14:1792661. doi: 10.3389/fpubh.2026.1792661 (PMC13493509; doi:10.3389/fpubh.2026.1792661)
Supplement: Supplementary file 11 [file Table_9.docx]

**Table S9. Schoenfeld residual test p-values for the proportional hazards assumption**

| Outcome |  | Model 1 | Model 2 | Model 3 | Model 4 |
| --- | --- | --- | --- | --- | --- |
| Surgery (4-year) | Before PSM | <0.001 | <0.001 | <0.001 | <0.001 |
|  | After PSM | <0.001 | <0.001 | <0.001 | <0.001 |
| Surgery (1-year) | Before PSM | <0.001 | <0.001 | <0.001 | <0.001 |
|  | After PSM | <0.001 | <0.001 | <0.001 | <0.001 |
| Opioid (including tramadol) | Before PSM | 0.014 | 0.032 | 0.072 | 0.006 |
|  | After PSM | 0.222 | 0.133 | 0.171 | 0.138 |
| Opioid (excluding tramadol) | Before PSM | <0.001 | <0.001 | <0.001 | <0.001 |
|  | After PSM | <0.001 | <0.001 | <0.001 | <0.001 |
